# Early-Stage State-of-Health Prediction of Lithium Batteries for Wireless Sensor Networks Using LSTM and a Single Exponential Degradation Model

**DOI:** 10.3390/s25072275

**Published:** 2025-04-03

**Authors:** Lorenzo Ciani, Cristian Garzon-Alfonso, Francesco Grasso, Gabriele Patrizi

**Affiliations:** Department of Information Engineering, University of Florence, Via di Santa Marta 3, 50139 Florence, Italy; cristiancamilo.garzonalfonso@unifi.it (C.G.-A.); francesco.grasso@unifi.it (F.G.); gabriele.patrizi@unifi.it (G.P.)

**Keywords:** machine learning, LSTM, recurrent neural networks, batteries’ state of health, NASA, single exponential model, prognostic and health management, remaining useful life, smart grids

## Abstract

**Highlights:**

**What are the main findings?**

- State-of-Health (SOH) battery prediction was tested under three different battery cycle consumption scenarios: 30%, 50%, and 65%.
- Training an LSTM model with only 50 records (equivalent to 30% of battery usage) enables accurate SOH prediction, achieving an MSE of $1.68\times 10^{-4}$ and an RMSE of $1.30\times 10^{-2}$. The best-performing model, trained with 110 records (65%), achieved an even lower MSE of $2.52\times 10^{-5}$ and an RMSE of $5.01\times 10^{-3}$.
- Two different training datasets were tested: one with raw sensor data (168 records) from NASA and another generated using a single exponential model (SEM) for curve fitting the battery degradation trend. Various LSTM architectures and hyperparameters were explored to optimize model performance.

**What is the implication of the main finding?**

- Accurate SOH estimation with limited data: the ability to achieve high accuracy with only 50 records suggests that SOH prediction can be achieved early in a battery’s life, allowing proactive maintenance and failure prevention.
- Improved energy management: reliable SOH prediction contributes to better decision-making in smart grids, optimizing energy storage and distribution.
- Cost reduction and extended battery life: enhanced SOH estimation minimizes maintenance costs and prevents premature battery replacements.

**Abstract:**

One of the most critical items from the reliability and the State-of-Health (SOH) point of view of wireless sensor networks is represented by lithium batteries. Predicting the SOH of batteries in sensor-equipped smart grids is crucial for optimizing energy management, preventing failures, and extending battery lifespan. Accurate SOH estimation enhances grid reliability, reduces maintenance costs, and facilitates the efficient integration of renewable energy sources. In this article, a solution for SOH prediction and the estimation of the Remaining Useful Life (RUL) of lithium batteries is presented. The approach was implemented and tested using two training datasets: the first consists of raw data provided by the Prognostics Center of Excellence at NASA, comprising 168 records, while the second is based on the curve fitting of the measured data using a single exponential degradation model. Long Short-Term Memory networks (LSTMs) were trained using data from three different scenarios, where battery cycle consumption reached 30%, 50%, and 65% correspondingly. Various architectures and hyperparameters were explored to optimize the models’ performance. The key finding is that training one of the models with only 50 records (equivalent to 30% of battery usage) enables accurate SOH prediction, achieving a Mean Squared Error (MSE) of $1.68\times 10^{-4}$ and Root Mean Squared Error (RMSE) of $1.30\times 10^{-2}$. The best model trained with 110 records achieved an MSE of $2.51\times 10^{-5}$ and an RMSE of $5.01\times 10^{-3}$.

## 1. Introduction

Smart energy grids are new solutions that use digital communication and automation to optimize the efficiency, reliability, and sustainability of energy distribution and consumption in the electrical grid [1]. Their significance is derived from multiple factors that contribute to the efficiency, sustainability, and resilience of energy systems, attaining carbon neutrality, and alleviating the effects of climate change [2]. Sensors play a crucial role in smart energy grids by providing real-time data on electricity consumption, grid performance, and environmental conditions. They help monitor voltage levels, detect faults [3], and optimize energy distribution, ensuring efficient and reliable power delivery. By integrating advanced sensor networks, smart grids can quickly respond to fluctuations in demand [4], reduce energy waste [5], and support renewable energy integration. This enhances overall grid stability, lowers operational costs, and improves sustainability. The fundamental components of smart energy grids are Energy Storage Systems (ESSs) [6]. Considering a continuous interchange of energy between production plants and smart users, the role of ESSs like lithium batteries and supercapacitors is crucial to ensure the stability, resilience, and efficiency of the smart energy grid infrastructure [7]. ESSs serve as pivotal components in balancing supply and demand dynamics, effectively managing fluctuations in renewable energy generation and consumption patterns.

By storing excess energy during periods of low demand and releasing it during peak demand times, energy storage facilities help optimize grid operations, mitigate grid instability, and enhance overall grid reliability [8]. Moreover, energy storage facilitates the integration of intermittent renewable energy sources, such as solar and wind, by providing a means to store excess energy for later use, thus contributing to the grid’s sustainability objectives and reducing dependency on fossil fuels. In essence, ESS is a hub in enabling the seamless transition towards a more resilient, sustainable, and responsive energy infrastructure for smart grids.

**Figure 1:**
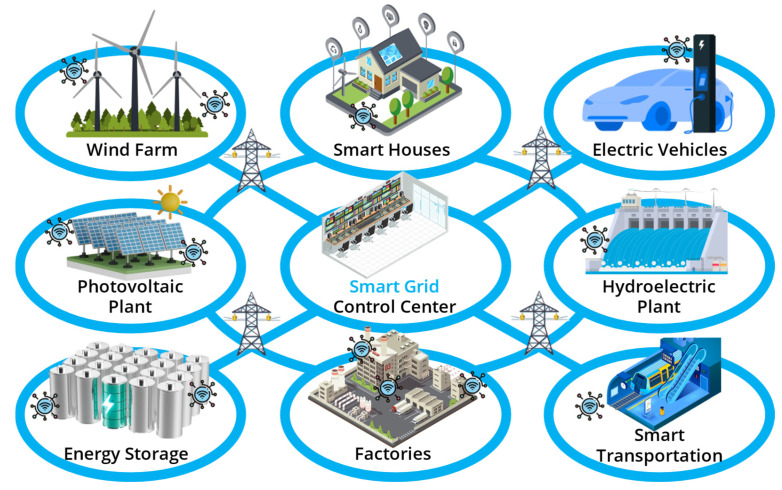
Example of a smart energy grid involving renewable production plants, energy storage systems, industry and transportation, smart consumers, and electric vehicles.

An overview of smart energy grids is presented in Figure 1, pointing out the essential role of batteries (and more generally of EESs) within the system, along with renewable energy sources like photovoltaic power plants, wind farms, and hydroelectric power plants. Figure 1 also highlights the presence of a wireless sensor network infrastructure, which is essential to monitor and manage all the different complex elements of the smart grid.

While extensively employed and continually advancing in various domains, persistent concerns remain about the rapid degradation processes associated with battery technologies [9,10,11]. In the context of a smart grid, the reliability and efficiency of the grid can be ensured only as long as the functionalities of the several wireless sensors are guaranteed. However, studies in the literature (see, for instance, [12]) pointed out that the main issues in the reliability analysis of smart wireless sensors are usually caused by battery degradation. Considering these concerns, the development of precise algorithms for estimating and forecasting the batteries’ State of Health (SOH) becomes a priority [13,14,15,16].

In this regard, Machine Learning (ML) [17,18,19] and Deep Learning (DL) [20] solutions are becoming the leading strategy in case of a battery’s SOH estimation. Such algorithms, when adeptly designed and implemented, enable real-time monitoring [21] and the assessment of the health status of batteries for wireless sensors in the grid [22]. This proactive approach facilitates timely interventions, minimizing the risk of safety incidents and ensuring the sustained performance of the entire smart grid infrastructure. Once the SOH has been estimated, it is possible to predict the Remaining Useful Life (RUL) [23] of the battery, which represents the remaining time interval before reaching the failure threshold [24]. Currently, common RUL forecasting methods for batteries include (but are not limited to) Kalman [25,26] and Particle Filters [27,28], Autoencoders [29], Support Vector Regression [30], Support Vector Machine [31,32], Artificial Neural Network [33], Recurrent Neural Network (RNN) [34,35,36], and many others. Most of the previously mentioned studies require a substantial amount of data for training to accurately learn patterns and make precise predictions. However, in real-world applications, such as wireless sensor networks, electric vehicles, and drones, continuously monitoring battery health is not always feasible due to constraints related to transmission bandwidth, available memory, and computational complexity. This study addresses this research gap by developing an ML model capable of accurately predicting battery capacity while requiring only a limited number of training records, ensuring efficiency and practicality in resource-constrained environments, such as the ones mentioned before.

This study provides an overview of ML algorithms incorporating RNNs, specifically Long Short-Term Memory (LSTM) networks, for battery health forecasting. The key contributions of this work include the following:

- Predicting battery degradation and estimating the RUL using a limited amount of data, specifically the initial aging cycles. This approach is particularly relevant in scenarios where continuous monitoring and storage of all critical battery parameters are not technically or economically feasible.
- Conducting a quantitative comparative analysis utilizing the NASA battery degradation dataset [37], evaluating model performance based on Mean Squared Error (MSE) and Root Mean Squared Error (RMSE). This analysis provides insights into the most effective ML models for SOH forecasting and RUL estimation.

## 2. Materials and Methods

### 2.1. Battery Degradation Dataset

To evaluate the performance of an RUL forecasting method, researchers [38] often rely on the publicly available battery degradation dataset provided by the Prognostics Center of Excellence at NASA [37]. This dataset is widely used because developing a comprehensive battery degradation dataset is a costly and time-consuming process that requires significant resources. The NASA dataset includes data from four batteries that were subjected to repeated charge and discharge cycles using a standard low-speed constant current profile. This profile closely represents real-world applications, such as those found in smart energy grids, making it highly relevant for predictive modeling. The key characteristics of the charge and discharge profile are outlined in Table 1. The rated capacity of each 18,650 battery cell under test is 2 Ah. The batteries are considered to have reached the end of their useful life when their capacity drops to 1.4 Ah, which corresponds to 70% of their original rated capacity.

In this study, multiple ML architectures are explored and evaluated for their effectiveness in predicting the SOH of batteries and estimating their RUL. A comprehensive comparison is conducted to assess the performance of these architectures under various operational scenarios, considering different levels of battery cycle consumption. By analyzing the strengths and limitations of each approach, this work aims to identify the most suitable ML models for accurate and reliable SOH prediction. The comparative study provides valuable insights into how different algorithms respond to varying amounts of training data, battery degradation patterns, and hyperparameter configurations. The scenarios used to train the models are as follows:

- Operating Scenarios:
  - *Early Prediction (EP):* In this case, the SOH prediction of the batteries was performed considering only the first 50 cycles of the battery life (which is approximately 30%) to train the models, and the remaining data were used for validation.
  - *Mid-Life Prediction (MLP):* In this case, the SOH prediction was performed after the batteries endured 80 cycles (which is approximately 48% of battery life). Remaining data were used for validation.
  - *Advanced Prediction (AP):* In this case, the SOH prediction of the batteries was conducted using only the first 110 cycles of battery life (approximately 65%) for model training, while the remaining cycles were used for validation.
  Even if in the context of this work the AP scenario is considered as a late prediction, it is worth mentioning that, compared to most ML and DL applications in the literature, the use of 65% of data for training (only 110 aging cycles in this application) is still considered to be an early or medium prediction time. So, the AP stands for advanced only compared to EP (50 cycles) and MLP (80 cycles), where all trainings are performed with a relatively low amount of data.

- Training Scenarios:
  - *Raw Data Training (RDT):* In this scenario, the training process is carried out by directly utilizing the raw data obtained from the NASA dataset, with a primary focus on battery capacity measurements. Instead of relying on pre-processed or derived features, the model is trained using the original capacity values recorded throughout the battery’s charge and discharge cycles. This can be carried out since the discharge capacity of the battery (i.e., the raw data included in the NASA dataset) is the main direct health indicator of the battery SOH and the main feature describing the battery degradation [24].
  - *Single Exponential Model (SEM):* In this case, training is not performed directly with the measured data. Instead, the raw data of the first 50 cycles *(EP scenario)*, 80 cycles *(MLP scenario)*, or 110 cycles *(AP scenario)* are processed using a curve-fitting toolbox to define the parameters of a single exponential degradation model as proposed in [36]. Then, training is performed using synthetic data obtained following the SEM in (1) with parameters reported in Table 2 in the case of battery B0007 of the NASA [37] dataset. The fitting has been performed with the Matlab R2024b “curve fitting toolbox” with the coefficient of determination *R*^2^ always above 0.97 for every operating scenario. In the following model, *Q*{*k*} is the synthetic dataset used for training, which represents the discharge capacity of the battery, *k* represents the cycle number, $C_{0}$ is the actual initial capacity of the battery, while $a_{z}$ and $b_{z}$ are the fitting parameters estimated at cycle k = z = 50 cycles in case of the EP scenario, k = z = 80 cycles in case of the MLP scenario, or k = z = 110 cycles in case of the AP scenario.

(1) Qk=C0+az*exp⁡bzk,

As a consequence of (1), the discharge capacity of the battery Q decreases when the number of charge/discharge cycles k increases (i.e., when the battery ages).

**Table 2:** Fitting parameters for training under the SEM scenario for battery B0007 of the NASA dataset.

Estimation Moment—*z*	$C_{0}$	$a_{z}$	$b_{z}$
50 cycles	1.8911 Ah	−0.2556	−51.55
80 cycles	1.8911 Ah	−1.0431	−110.59
110 cycles	1.8911 Ah	−0.8722	−99.92

### 2.2. ML Component

**Figure 2:**
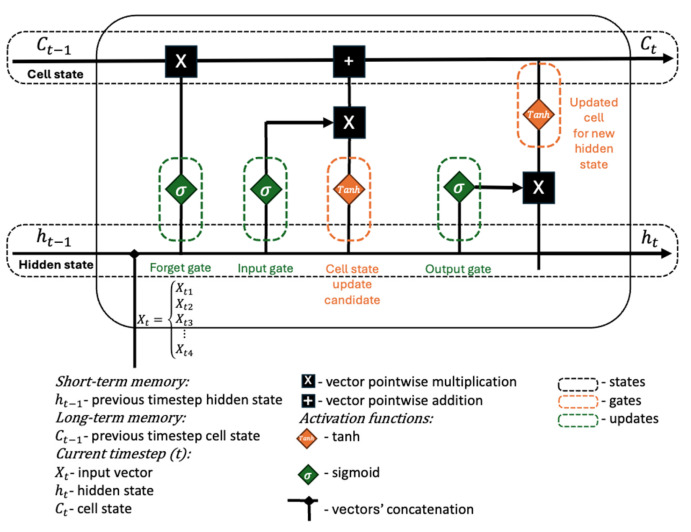
LSTM architecture with all the components detailed.

The ML implementation is based on an LSTM network. The LSTM model was selected following a comparison with the Autoregressive Integrated Moving Average (ARIMA) model. ARIMA demonstrated limitations in handling nonlinear degradation trends, as it does not dynamically adapt to evolving degradation patterns and requires periodic retraining, particularly when dealing with non-stationary datasets, as discussed in [39,40]. In the domain of NNs, LSTMs are specifically designed and optimized for sequential time-series prediction, making them a more suitable choice for accurately modeling the complex degradation behavior of lithium-ion batteries. LSTMs are particularly well-suited for time series forecasting tasks due to their ability to retain long-term dependencies in sequential data. This capability enables the model to effectively capture patterns in battery degradation over time, improving the accuracy of SOH prediction and RUL estimation. Figure 2 provides a complete representation of the LSTM architecture based on the first time that the concept was introduced [41]. The cell state ($C_{t}$) acts as the long-term memory of the network, preserving relevant information across time steps while being selectively updated by the gates. The ($h_{t}$) represents the short-term memory and serves as the output and input of the LSTM at each timestep, influencing both the next hidden state and the final prediction. The previous timestep hidden state $\left(C_{t-1}\right)$ and previous timestep cell state $\left(h_{t-1}\right)$ provide historical context by carrying forward information from past inputs. The input vector ($x_{t})$ represents the current timestep’s data and is concatenated with the previous hidden state $\left(h_{t-1}\right)$ to compute gate activations. Operations such as vector pointwise multiplication and vector pointwise addition regulate how much past and new information should be retained or discarded in the cell state. The tanh activation function is used to scale values within a range of [−1, 1], ensuring stable gradient propagation, while the sigmoid activation function outputs values between 0 and 1 to control the degree of information flow in the gates. Lastly, vector concatenation combines the input vector and previous hidden state, allowing the model to process both current and past information simultaneously. Together, these elements enable LSTMs to capture long-term dependencies in sequential data, making them well-suited for applications such as battery SOH prediction and RUL estimation. In an LSTM, the states (black dotted lines), gates (orange dotted lines), and updates (green dotted lines) work together to regulate information flow and maintain long-term dependencies in sequential data. The cell state serves as the memory unit, preserving relevant information across time steps, while the hidden state acts as the short-term representation that influences both the next hidden state and the final output. The gates control the flow of information.

The forget gate decides how much past information should be discarded from the cell state, the input gate determines what new information should be added, and the output gate regulates what part of the cell state contributes to the hidden state. Through updates, the LSTM refines its memory, selectively forgetting, retaining, or passing information to optimize learning.

#### 2.2.1. Architectural Compositions

**Figure 3:**
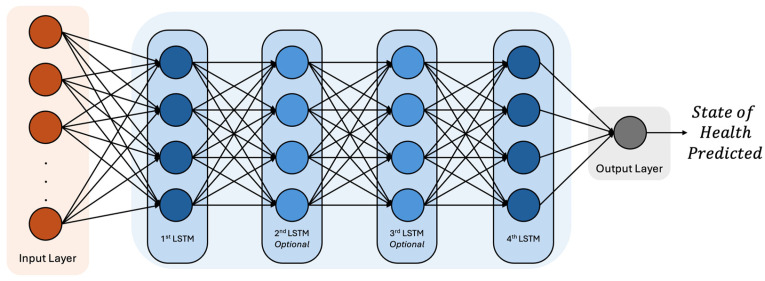
LSTM architectures tested to evaluate the performance through the different options.

One of the main strategies implemented is the evaluation of different training scenarios by testing the various architectural compositions of LSTM-based models, as illustrated in Figure 3. The experimental setup consists of three distinct architectures:

- Simple: includes two LSTM layers, the first and the fourth ones.
- Medium: incorporates three LSTM layers, the first, second, and fourth ones.
- Complex: consists of four LSTM layers.

Regardless of the configuration, the first and last layers remain fixed in the architecture, while the second and third layers are optional, depending on the model variant being tested. The rationale behind this approach is to systematically analyze the impact of increasing model complexity on prediction accuracy and computational efficiency. By implementing and evaluating these different architectures, the goal is to identify the most suitable model for accurately predicting the SOH of batteries while maintaining an optimal balance between performance and computational cost. This methodology allows for a structured comparison, ensuring that the selected architecture effectively captures the underlying degradation patterns of the batteries while avoiding unnecessary overfitting or excessive resource consumption.

**Figure 4:**
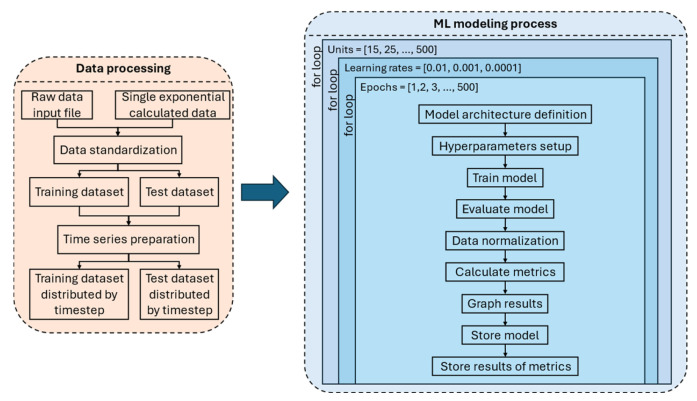
Algorithm complete pipeline implemented with hyperparameters.

To further enhance model performance and achieve the highest possible accuracy in SOH prediction, various hyperparameter configurations were tested across the different LSTM architectures previously described. The complete workflow of this process is illustrated in Figure 4, which is divided into two main stages: data processing and ML modeling.

#### 2.2.2. Data Processing

The data processing pipeline begins with two possible input data sources. The first option is using raw data directly from the NASA dataset, while the second option involves using a SEM-computed dataset, where values are generated based on the model defined in Equation (1). Once the input data are selected, the next step involves standardization and normalization to ensure that all values remain within the same numerical range, typically between 0 and 1. Those processes are applied to both NASA and SEM datasets. This step is crucial for stabilizing the training process and improving convergence. After normalization, the dataset is split into training and test sets to enable effective model training and evaluation.

Following data preparation, the ML modeling process begins with formatting the data into a structured input suitable for LSTM training. One of the critical aspects of this step is the definition of the time step parameter, which determines the number of past observations (records) included in each training instance. This configuration is essential for allowing the LSTM to capture temporal dependencies, as it enables the model to learn how past values influence the current SOH state. By incorporating historical records into each training instance, the LSTM can identify long-term patterns and relationships in battery degradation, leading to more accurate predictions.

#### 2.2.3. ML Modeling

Once the training and test datasets are prepared, the ML modeling process can begin. The first step to focus on is to optimize the hyperparameters to achieve the best possible performance, stability, and generalization ability for predicting the SOH of batteries, which directly influences the estimation of the RUL, and it is also essential for efficient battery management in smart grids. A key hyperparameter in this process is the number of units (neurons) in each LSTM layer, which defines the model’s capacity to learn and retain complex temporal dependencies. Increasing the number of units allows the model to capture more intricate patterns in the data, making it highly effective in modeling long-term dependencies within battery degradation trends. However, an excessive number of units can result in overfitting, where the model becomes too specialized in the training data and loses its ability to generalize well to unseen data, leading to poor real-world performance. On the other hand, an insufficient number of units may cause underfitting, where the model is unable to learn meaningful patterns from the data, resulting in low predictive accuracy. Therefore, selecting an optimal number of units is crucial for balancing the model’s complexity and generalization capacity, ensuring that it effectively captures battery degradation behaviors without compromising predictive accuracy.

The learning rate is a fundamental hyperparameter in the training process of an LSTM network, as it controls the magnitude of weight adjustments at each training step based on the computed error. It plays a critical role in determining the speed and stability of model convergence. A higher learning rate accelerates the convergence process, enabling the model to reach an optimal solution faster. However, it also increases the risk of overshooting the optimal solution, causing instability and potential divergence. In contrast, a lower learning rate ensures gradual and precise weight updates, allowing the model to refine its predictions incrementally. However, if set too low, it may lead to excessively prolonged training times or cause the model to become trapped in local minima, preventing it from achieving the best possible performance. To systematically analyze the impact of this parameter, three different learning rates were tested in this study: 0.01, 0.001, and 0.0001, enabling a comparative evaluation of their effects on model convergence and predictive accuracy.

Another crucial hyperparameter in LSTM training is the number of epochs, which represents the number of complete passes through the training dataset. A higher number of epochs allows the model to learn more effectively from the data, refining its internal representations and improving performance. However, training for too many epochs can lead to overfitting, where the model becomes too closely adapted to the training data and loses its ability to generalize to new, unseen data. Conversely, training for too few epochs may result in an underfitted model that has not fully captured the complex temporal dependencies necessary for accurate SOH prediction. To optimize training duration while preventing overfitting, models were trained for a maximum of 500 epochs. However, to improve computational efficiency and prevent unnecessary training, an early stopping criterion was applied: training was terminated once the loss function in the training phase dropped below 1 × 10^−5^, indicating that the error was minimal and further training iterations would not result in significant performance improvements. Effectively balancing these hyperparameters, number of units, learning rate, and number of epochs is essential for achieving an accurate and generalizable LSTM model. By carefully tuning these parameters, the model can efficiently extract meaningful patterns from historical battery degradation data, adapt to complex temporal dependencies, and generate highly precise predictions of battery SOH and RUL. This systematic approach ensures that the final model is not only computationally efficient but also capable of delivering reliable and robust predictions for real-world applications in smart energy grids.

Once the hyperparameters have been carefully defined and optimized, the modeling process proceeds through a structured sequence of steps to ensure thorough evaluation, accurate performance assessment, and reproducibility of the results. The first step involves training the model using the selected dataset, where the LSTM network learns from historical battery degradation patterns by adjusting its internal weights based on the provided input data and the calculated loss function. Once training is completed, the next stage is evaluating the model’s performance, which involves using the trained network to make predictions on the test dataset and comparing the predicted values with the actual values to assess accuracy. Following this, performance metrics such as MSE and RMSE are computed to quantify the model’s predictive accuracy and generalization ability. Subsequently, data normalization is applied to ensure that all numerical inputs remain within a consistent range, improving the model’s stability and convergence behavior. After the normalization process, the model’s predictions and calculated error metrics are graphically represented through visualizations such as loss curves and SOH prediction graphs. These visualizations facilitate an easier understanding of the model’s behavior and provide insights into potential areas of improvement. The next step involves storing the trained model, allowing it to be rebuilt, fine-tuned, and reused in the future without the need for retraining from scratch. Finally, to maintain a systematic record of the results, all obtained metrics, model configurations, and generated predictions are stored in a structured file, enabling a comprehensive tracking system for comparing different model versions and training scenarios. This logging process not only facilitates reproducibility and further model improvements but also ensures transparency in performance evaluation. By following this structured pipeline, the proposed methodology ensures that the LSTM-based SOH prediction model is trained, validated, and stored in an efficient manner, ultimately contributing to more reliable and scalable battery health forecasting systems.

## 3. Results and Discussion

In this section, the performance of the trained LSTM-based models is analyzed, considering different architectural configurations and hyperparameter selections. The results demonstrate that the model successfully captures the temporal dependencies in battery degradation, leading to accurate SOH estimation and RUL predictions. Moreover, graphical representations of the predicted values obtained after training with NASA’s raw and SEM datasets versus actual SOH values show that the model follows the degradation curve with minimal deviation, validating its robustness. These findings underscore the potential of LSTM networks in battery health prediction applications and highlight the importance of hyperparameter optimization in enhancing predictive performance.

### 3.1. Metrics

To evaluate the predictive accuracy and performance of the proposed LSTM-based SOH prediction model, two key error metrics were analyzed: MSE and RMSE. These metrics provide a quantitative assessment of the difference between the predicted and actual SOH values, offering insights into the model’s precision and generalization ability. MSE measures the average squared error, penalizing larger deviations more heavily, while RMSE, as the square root of MSE, provides an interpretable error magnitude. A lower value for both metrics indicates a more accurate prediction, ensuring the reliability of the model in forecasting battery degradation trends.

#### 3.1.1. Mean Squared Error (MSE)

It is a fundamental metric used to evaluate the performance of predictive models, particularly in regression tasks. It is defined as the average of the squared differences between the actual and predicted values, mathematically expressed as (2):

(2) $$MSE=\frac{1}{n}\;\sum_{i=1}^{n}{\left(y_{i}-{\hat{y}}_{i}\right)}^{2},$$

where $y_{i}$ represents the actual SOH values, ${\hat{y}}_{i}$ denotes the predicted SOH values, and n is the total number of observations. By squaring the errors, MSE places a greater penalty on larger deviations, making it particularly sensitive to outliers. This characteristic ensures that the model prioritizes minimizing significant prediction errors, leading to more reliable and precise SOH estimations. In the context of battery health monitoring, a lower MSE value indicates a higher accuracy of the LSTM model, reflecting its ability to effectively capture battery degradation trends and support informed decision-making in smart energy grid applications.

#### 3.1.2. Root Mean Squared Error (RMSE)

The RMSE is a widely used metric for assessing the accuracy of predictive models, particularly in a regression. The RMSE is derived from the MSE by taking its square root. Mathematically, the RMSE is defined as (3):

(3) $$RMSE=\sqrt{\frac{1}{n}\;\sum_{i=1}^{n}{\left(y_{i}-{\hat{y}}_{i}\right)}^{2}},$$

where $y_{i}$ represents the actual SOH values, ${\hat{y}}_{i}$ denotes the predicted SOH values, and n is the total number of observations. RMSE provides a measure of the average magnitude of prediction errors, giving greater weight to larger deviations due to the squaring operation. This makes RMSE particularly useful for evaluating models where significant errors must be minimized. A lower RMSE value indicates better model performance, reflecting its ability to generalize well to unseen data and accurately track battery degradation trends. By combining RMSE with other evaluation metrics, a more comprehensive assessment of the model’s predictive capabilities can be achieved, ensuring its reliability in real-world applications.

### 3.2. Comparative Analysis

In this section, a detailed analysis of the results obtained from the comparative evaluation of different ML algorithms and various experimental scenarios, as described in Section 2, is presented. The primary objective of this comparative study is to assess the effectiveness of different ML-based approaches in predicting the SOH and estimating the RUL of lithium-ion batteries under diverse operating conditions. The analysis provides valuable insights into the performance, computational efficiency, and accuracy of each method in order to determine the most suitable model for battery health forecasting. The selection of the best-performing models was conducted by identifying those that achieved the lowest values in the MSE and RMSE metrics. These performance indicators were used as primary evaluation criteria to ensure the models exhibited the highest accuracy in predicting the SOH and RUL of the lithium-ion batteries. To illustrate the evaluation process, battery B0007 from the dataset [37] has been selected as a representative example. Table 3 presents the results obtained by varying the number of LSTM layers and the quantity of training records while keeping the number of units and learning rate constant across all tested configurations. Based on the evaluation metrics, the best-performing model for each hyperparameter combination was selected as the optimal configuration for that specific scenario. Consequently, the best model from each set of hyperparameter combinations was included in Table 4, following this selection process.

Table 4 provides a comprehensive summary of the best metric results obtained after systematically evaluating all possible combinations of hyperparameters for each type of LSTM architecture tested in this study. The architectures considered include the simple, medium, and complex configurations, each varying in the number of layers and overall structural complexity. Additionally, the results are presented based on different quantities of training samples, allowing for a detailed analysis of how the amount of available data influences model performance. The hyperparameters tested in this evaluation, as previously detailed in Section 2.2, include key factors such as the number of units per layer, the learning rate, and the number of epochs. By systematically exploring various hyperparameter settings across different architectures and training sample sizes, this analysis aims to identify the most effective model configuration for accurately predicting the SOH of the battery.

**Figure 5:**
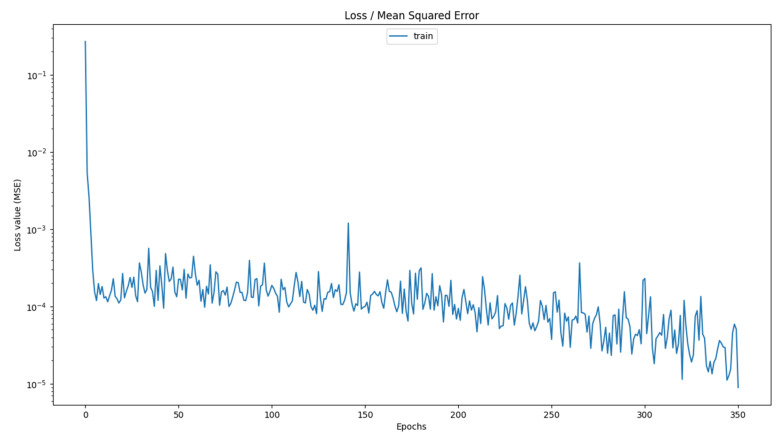
Loss function of a model trained with three LSTM layers under the MLP operating scenario, where training was halted after 351 epochs due to the early stopping condition being fulfilled.

After analyzing the results, a detailed discussion regarding the operating scenario with EP can be conducted. It was observed that the best predictive performance was achieved when using the minimum quantity of training samples, which in this case consisted of 50 records. This finding highlights the potential of achieving accurate SOH estimation with a reduced dataset, which is particularly beneficial in practical applications where data collection may be limited or costly. Furthermore, across all scenarios that give the best performance, the optimal architecture consisted of only two LSTM memory layers. Additionally, it was found that the most effective models were those configured with a relatively small number of units per layer, reinforcing the idea that an excessively large network does not always yield superior results. Another critical observation from the results was that some of the best performing models do not require the full 500 epochs to converge. An example is shown in Figure 5 in the case of the MLP scenario with three LSTM layers trained with SE (the Y axis is on a logarithmic scale).

The same methodology described previously was applied to the MLP and AP scenarios to analyze model performance across different stages of battery degradation. In the case of MLP, it was observed that most of the best-performing models were obtained using four LSTM layers. Additionally, the optimal learning rate remained consistent at 0.001 across all tested configurations. All the models in this scenario required the full 500 epochs for training. Another notable observation is that the number of units per layer remained relatively small in most cases, with only one exception where a higher number of units was used. For the AP scenario, the best results were achieved using two LSTM layers, while the learning rate remained unchanged at 0.001, like the MLP case. A significant finding in this scenario is that all models demonstrated convergence and started to fit the data within fewer than 500 epochs. To ensure a consistent and equidistant selection of training records across the different prediction scenarios, the final chosen configurations corresponded to 50 records for EP, 80 records for MLP, and 110 records for AP. This structured approach allowed for a comprehensive comparison of performance at different battery life stages.

**Figure 6:**
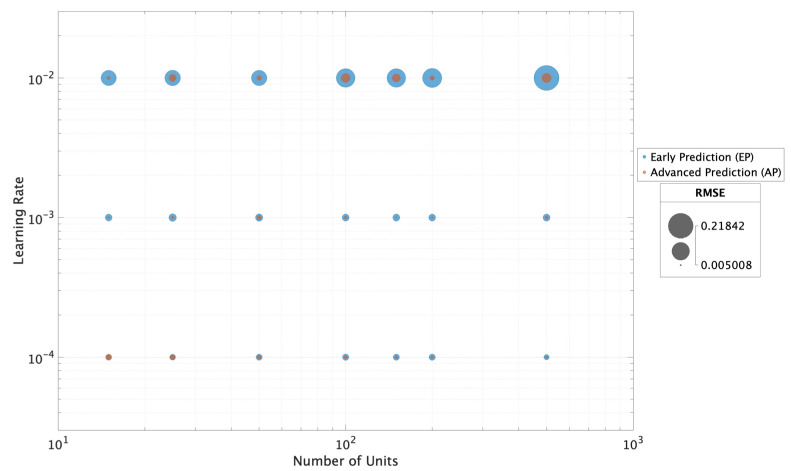
Relationship between the learning rate and the number of epochs, illustrating their impact on the RMSE metric in the EP and AP operating scenarios with the SE training datasets.

Figure 6 illustrates the relationship between different learning rates (0.01, 0.001, 0.0001) and the number of units (15, 25, 50, 100, 150, 200, 500) tested in the model training process. The size of each data point represents the RMSE metric obtained after testing, where larger points indicate higher RMSE values and lower performance, while smaller points correspond to better models. This graph was generated using all the results obtained from tests made in the EP and AP operating scenarios, where models were trained with 50 and 110 records, represented in blue and orange, respectively. The graph shows that the EP scenario exhibits a higher RMSE, likely due to the smaller amount of training data used. However, it is important to note that for models with 15, 25, and 50 units and a learning rate of 0.0001, the RMSE values were similar across both scenarios, indicating that the quantity of training records had minimal impact under these specific conditions.

The EP scenario explained before in Section 2.1 is specifically considered for this assessment. As part of this analysis, Table 5 presents a comprehensive summary of the key performance metrics for comparison according to the RDT and SEM scenarios for each of the LSTM architectures tested. These metrics serve as critical indicators of the model’s accuracy and reliability, allowing for a thorough comparison of the different approaches.

**Figure 7:**
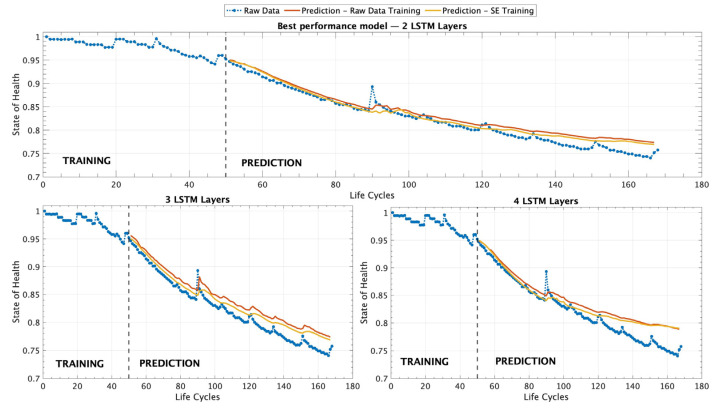
Results for battery B0007 using three different ML architectures under the EP scenario. The models were trained with 50 records, considering both the RDT and SEM training approaches.

Table 5 provides a comparison of the performance of different models trained under varying conditions across all scenarios, including the simple, medium, and complex architectures. From the results presented, it is evident that the models trained using the SEM approach demonstrate superior performance when compared to those trained only on RDT data. This observation is consistent across all experimental setups, highlighting the advantage of incorporating a pre-processed degradation representation for improved predictive accuracy in battery SOH estimation. The SEM, by capturing the underlying degradation trend in a more structured manner, enhances the learning process of the ML models, leading to lower error metrics and a more reliable prediction capability. Another particularly noteworthy finding from the comparative analysis is that the simplest model architecture, which consists of only two LSTM layers, outperforms the more complex models in terms of predictive accuracy and overall performance. Despite the expectation that deeper architectures with additional layers might enhance the model’s ability to capture intricate dependencies in battery degradation patterns, the results indicate that increasing model complexity does not necessarily lead to better outcomes. Instead, the additional layers in the medium and complex architectures may introduce unnecessary complexity, leading to potential overfitting or difficulties in optimizing the learning process. This finding underscores the importance of selecting an appropriate model complexity to achieve optimal performance, rather than if larger and more sophisticated architectures inherently yield superior results. Figure 7 provides a comprehensive visualization of all the different experimental scenarios that were tested using the different ML models trained with a dataset containing only 50 records as an EP model.

Figure 7 presents a set of three graphical representations that illustrate the predictive performance of the different LSTM architectures tested in this study. The first graph, on top, corresponds to the model that demonstrated the best overall performance, which is composed of two LSTM layers. This architecture outperformed the other configurations in terms of accuracy and predictive capability, as evidenced by the evaluation metrics reported in Table 5. The superior performance of this model suggests that a simpler structure with two layers is sufficient to capture the underlying patterns of battery degradation effectively. The second and third graphs in Figure 7 correspond to the results obtained from models with three and four LSTM layers, respectively. However, the results indicate that while these deeper architectures were able to learn from the training data, they did not surpass the accuracy achieved by the two-layer model. The comparative analysis presented in Figure 7 highlights the importance of selecting an optimal model architecture and hyperparameters that balance complexity and the generalization capability, ensuring that the predictive model remains both accurate and efficient for battery SOH estimation.

Table 6 presents the results obtained from the models trained using a dataset consisting of 80 records MLP, allowing for a more extensive learning process compared with the previous scenario with 50 training samples EP. The table provides a detailed comparison of the predictive performance of each tested architecture, highlighting key evaluation metrics that assess the accuracy and reliability of the SOH estimation. The inclusion of multiple architectures in the evaluation ensures a comprehensive understanding of the relationship between the number of layers, the network depth, and the overall model performance. Furthermore, the results contribute to determining the most effective model configuration for battery degradation forecasting.

**Figure 8:**
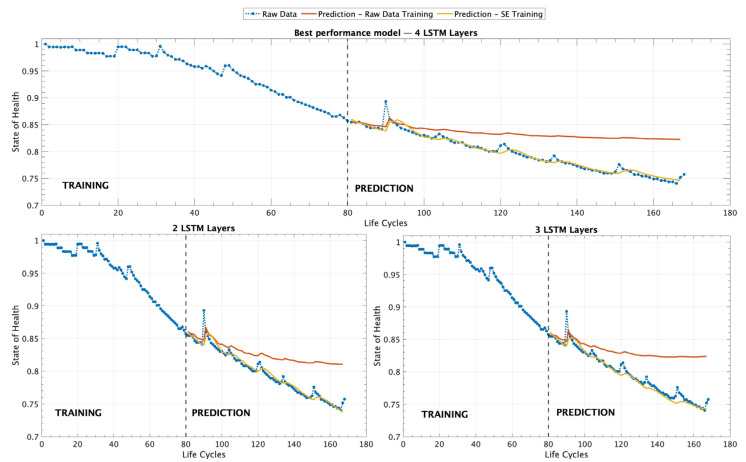
Results for battery B0007 using three different LSTM architectures under the MLP scenario. The models were trained with 80 records, utilizing both the RDT and SEM training approaches.

Table 6 provides a clear comparison of the performance between the SEM and RDT training scenarios, demonstrating that the models trained with the SEM consistently achieve significantly better results. In this case, the most complex model, consisting of four LSTM layers, yields the lowest MSE and RMSE, indicating superior predictive accuracy and robustness in battery SOH estimation. Figure 8 provides a detailed visualization of the different experimental scenarios analyzed, serving as a complement to Table 6 by graphically representing the results. It illustrates the performance of different ML models trained with a dataset consisting of 80 records within the MLP operating scenario.

Figure 8 presents three different graphs that illustrate the performance of different ML models tested under the MLP scenario. The first graph, on top, corresponds to the model composed of four LSTM layers (complex), which demonstrated the best overall performance according to the evaluation metrics presented in Table 6. This model exhibited the lowest MSE and RMSE, indicating its superior ability to accurately predict the SOH of the battery. The second and third graphs, located on the left and right sides of the figure, respectively, display the results obtained from models utilizing two and three LSTM layers. Table 7 presents the results obtained for the various architectural models trained under the AP operating scenario, which was made using a dataset consisting of 110 records.

**Figure 9:**
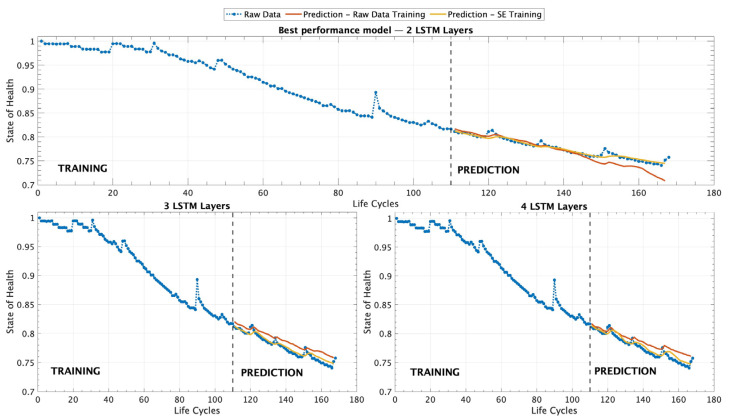
Performance of battery B0007 across three distinct LSTM architectures within the AP scenario. The models were trained using a dataset of 110 records.

Table 7 presents a comprehensive comparison of the performance between the SEM and RDT training scenarios, highlighting that models trained using the SEM approach consistently achieve superior results. Notably, in this case, the simplest model, composed of two LSTM layers, demonstrates the best metrics, indicating enhanced predictive accuracy and robustness in estimating the SOH of the battery. To further illustrate these findings, Figure 9 provides a detailed graphical representation of the different experimental scenarios analyzed. This figure serves as a visual complement to Table 7, offering an intuitive depiction of the performance of various LSTM architecture models trained with a dataset containing 110 records within the AP operating scenario.

**Figure 10:**
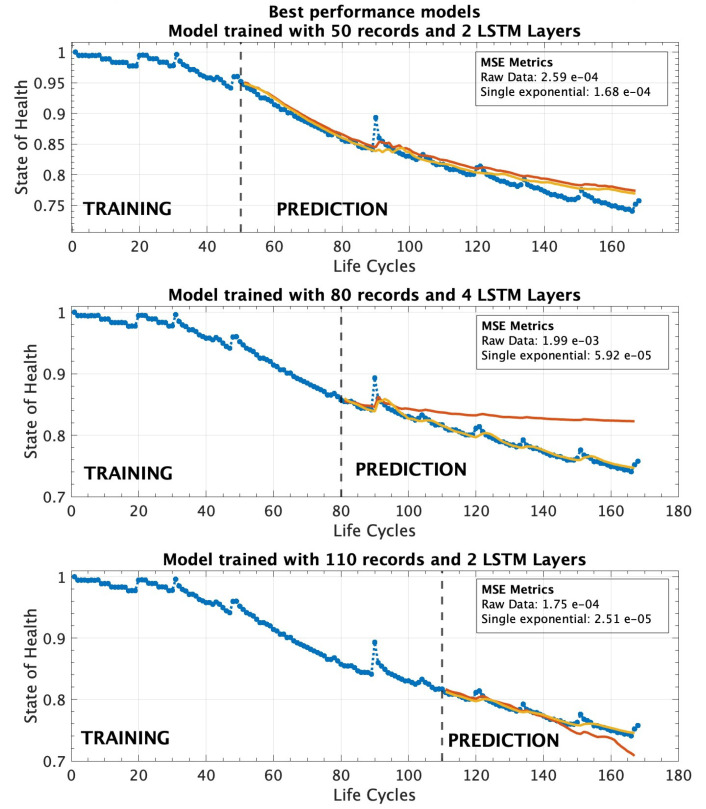
Best performing model for each operational scenario at 30%, 48%, and 65% of the battery’s lifetime, comparing results from both the RDT and SEM training approaches.

Figure 9 displays three graphs depicting the performance of the different models evaluated under the AP scenario. The top graph represents the model with two LSTM layers, which achieved the best performance based on the evaluation metrics in Table 7. The graphs on the left and right illustrate the results obtained from models with three and four LSTM layers, respectively, providing a comparative view of their performance in estimating the state of health of the battery. Figure 10 presents a comparative visualization of the best-performing models from each operational scenario. The EP scenario is displayed at the top, followed by the MLP scenario in the middle, and the AP scenario at the bottom. Across all scenarios, models trained using the SEM consistently outperform those trained with RDT, demonstrating superior predictive accuracy and stability. When considering only the evaluation metrics, the AP scenario, trained with the largest dataset of 110 records to predict approximately 60 future cycles, exhibits the most accurate performance. However, the MLP scenario, trained with only 80 records, also demonstrates highly reliable predictive behavior. In this scenario, it is important to highlight that the model trained with raw experimental data exhibited suboptimal performance. This outcome may be attributed to several factors, including the presence of higher noise levels in the data acquisition process, as it is derived from real-world conditions. Additionally, unaccounted variability within the dataset, such as fluctuations caused by environmental conditions, diverse operational scenarios, or inconsistencies in battery manufacturing, could further impact the model’s ability to generalize effectively. Additionally, the EP scenario, which corresponds to approximately 30 percent of the battery’s lifespan, provides a strong SOH prediction, highlighting the robustness of the proposed approach across different training conditions. As expected, the performance of the EP scenario, which was trained with 50 records, is weaker compared with the MLP and AP scenarios. The reduced amount of training data likely constrained the model’s ability to capture the underlying degradation patterns effectively, leading to higher MSE and RMSE values.

### 3.3. Performance Comparison with Related Work

This section compares the results obtained in this study with those reported in the existing literature summarized in Table 8. In [42], a comparative analysis was conducted using ANN, LSTM networks, Support Vector Machines (SVM), Gaussian Process Regression (GPR), and Hierarchical Relevance Vector Machines (H-RVM). These models were trained using data from the first 50 cycles and then used to predict the remaining SOH of the battery. Consequently, our EP scenario can be directly compared with the results presented in that study. Similarly, in [43], a comparison was performed among Symbolic Regression (SR), eXtreme Gradient Boosting (XGBoost), Linear Neural Networks (Linear NN), Lasso Regression, Ridge Regression (Lasso), ElasticNet, and GPR. In this case, the models were trained on the first 80 cycles and used to predict the remaining battery life. Therefore, the results obtained in our MLP scenario can be compared with those reported in that study. Both studies report the RMSE metric, enabling a direct comparison with our findings. For the AP scenario, no related studies were identified at the 110-cycle battery life point for direct comparison.

### 3.4. Challenges and Potential Improvements

One of the challenges encountered in this study was determining the appropriate number of rows required to effectively split the dataset for training and testing the models. Regarding the limitations and potential improvements, a key consideration is the generalizability of the proposed ML models from this paper. Future work could involve testing these models on additional battery datasets from the NASA repository to further evaluate their performance and robustness, as the current study was conducted exclusively using data from the “B0007” battery, also with a dataset generated from other experimental laboratories using batteries with similar characteristics.

## 4. Conclusions

This study presents the development and evaluation of ML models based on LSTM networks for predicting the SOH of batteries and estimating their RUL. The findings highlight the effectiveness of these models in providing accurate predictions, which can assist battery users in determining the optimal time for replacement, ultimately enhancing reliability and operational efficiency.

The results presented in the tables and graphs demonstrate that, in all cases, the models trained using the SEM consistently outperform those trained with the RDT. This superior performance is reflected in the evaluation metrics, indicating that the SEM-based approach enhances the predictive accuracy and reliability of the developed models for battery SOH estimation and RUL prediction.

This study demonstrates that more complex ML architectures do not always yield better results. In the case of EP and AP scenarios, the most effective models were those with a simpler architecture consisting of only two LSTM layers. These findings highlight the importance of selecting an appropriate model complexity to balance predictive performance and computational efficiency, rather than assuming that deeper networks will always provide superior outcomes.

The model trained with 110 records achieved the lowest MSR, reaching a value of $2.51\times 10^{-5}$, demonstrating its high predictive accuracy. However, the models trained with 80 and 50 records also exhibited strong performance, obtaining MSE values of $5.92\times 10^{-5}$ and $1.68\times 10^{-4}$, respectively. These results indicate that even with a reduced number of training samples, the models maintained a high level of accuracy, highlighting the efficiency of the proposed approach in predicting the SOH of the battery.

## Figures and Tables

**Table 1:** Features of the charge/discharge profile for the considered battery degradation dataset.

Charge		Discharge	
Profile	CC-CV	Profile	CC
Constant Current	1.5 A	C-Rate	1
Constant Voltage	4.2 V	Cut-off Voltage	2.2 ÷ 2.7 V

**Table 3:** Results obtained from evaluating one hyperparameter configuration for the EP scenario using models trained with 50, 60, and 70 records for battery B0007.

LSTMs	Training Samples	Units	Learning Rate	Epochs	MSE	RMSE
2	50	15	0.0001	500	$1.68\times 10^{-4}$	$1.30\times 10^{-2}$
2	60	15	0.0001	500	$2.59\times 10^{-4}$	$1.61\times 10^{-2}$
2	70	15	0.0001	500	$2.58\times 10^{-4}$	$1.61\times 10^{-2}$
3	50	15	0.0001	500	$4.84\times 10^{-4}$	$2.20\times 10^{-2}$
3	60	15	0.0001	500	$4.14\times 10^{-4}$	$2.04\times 10^{-2}$
3	70	15	0.0001	500	$9.38\times 10^{-4}$	$3.06\times 10^{-2}$
4	50	15	0.0001	500	$4.64\times 10^{-4}$	$2.16\times 10^{-2}$
4	60	15	0.0001	500	$8.62\times 10^{-4}$	$2.94\times 10^{-2}$
4	70	15	0.0001	500	$1.64\times 10^{-3}$	$4.05\times 10^{-2}$

**Table 4:** Summary of the best results obtained after evaluating all possible combinations of model configurations with hyperparameters for each quantity of training samples used for battery B0007.

LSTMs	Operating Scenario	Training Samples	Units	Learning Rate	Epochs	MSE	RMSE
2	Early Prediction (EP)	50	15	0.0001	500	$1.68\times 10^{-4}$	$1.30\times 10^{-2}$
2	Early Prediction (EP)	60	50	0.0001	500	$2.40\times 10^{-4}$	$1.55\times 10^{-2}$
2	Early Prediction (EP)	70	25	0.01	500	$2.43\times 10^{-4}$	$1.56\times 10^{-2}$
4	Mid-Life Prediction (MLP)	80	50	0.001	500	$5.92\times 10^{-5}$	$7.69\times 10^{-3}$
4	Mid-Life Prediction (MLP)	85	150	0.001	500	$7.81\times 10^{-5}$	$8.84\times 10^{-3}$
4	Mid-Life Prediction (MLP)	90	25	0.001	500	$3.69\times 10^{-5}$	$6.08\times 10^{-3}$
3	Mid-Life Prediction (MLP)	95	25	0.001	351	$3.00\times 10^{-5}$	$5.48\times 10^{-3}$
3	Advanced Prediction (AP)	100	50	0.001	416	$2.86\times 10^{-5}$	$5.34\times 10^{-3}$
2	Advanced Prediction (AP)	105	25	0.001	367	$2.79\times 10^{-5}$	$5.28\times 10^{-3}$
2	Advanced Prediction (AP)	110	150	0.001	455	$2.51\times 10^{-5}$	$5.01\times 10^{-3}$
2	Advanced Prediction (AP)	120	100	0.001	429	$2.57\times 10^{-5}$	$5.07\times 10^{-3}$

**Table 5:** Evaluation of battery B0007 utilizing three distinct ML architectures for EP, trained with 50 records in both RDT and SEM training scenarios.

Algorithm	Raw Data Training—RDT Scenario		Single Exponential Model—SEM Scenario	
	MSE	RMSE	MSE	RMSE
Simple	$2.59\times 10^{-4}$	$1.61\times 10^{-2}$	$1.68\times 10^{-4}$	$1.30\times 10^{-2}$
Medium	$5.25\times 10^{-4}$	$2.29\times 10^{-2}$	$2.72\times 10^{-4}$	$1.65\times 10^{-2}$
Complex	$5.67\times 10^{-4}$	$2.38\times 10^{-2}$	$4.64\times 10^{-4}$	$2.16\times 10^{-2}$

**Table 6:** Comparative analysis of battery B0007 using three different ML architectures for MLP, trained with 80 records under the RDT and SEM training scenarios.

Algorithm	Raw Data Training—RDT Scenario		Single Exponential Model—SEM Scenario	
	MSE	RMSE	MSE	RMSE
Simple	$1.33\times 10^{-3}$	$3.64\times 10^{-2}$	$6.02\times 10^{-5}$	$7.76\times 10^{-3}$
Medium	$1.85\times 10^{-3}$	$4.30\times 10^{-2}$	$7.21\times 10^{-5}$	$8.49\times 10^{-3}$
Complex	$1.99\times 10^{-3}$	$4.46\times 10^{-2}$	$5.92\times 10^{-5}$	$7.69\times 10^{-3}$

**Table 7:** Correlative analysis of battery B0007 performance using three different LSTM architectures for the AP scenario, trained with 110 records under both the RDT and SEM training approaches.

Algorithm	Raw Data Training—RDT Scenario		Single Exponential Model—SEM Scenario	
	MSE	RMSE	MSE	RMSE
Simple	$1.75\times 10^{-4}$	$1.32\times 10^{-2}$	$2.51\times 10^{-5}$	$5.01\times 10^{-3}$
Medium	$1.57\times 10^{-4}$	$1.25\times 10^{-2}$	$3.03\times 10^{-5}$	$5.50\times 10^{-3}$
Complex	$1.47\times 10^{-4}$	$1.21\times 10^{-2}$	$3.64\times 10^{-5}$	$6.04\times 10^{-3}$

**Table 8:** A comparison between results obtained in previous studies and the proposed solution in this study.

Operating Scenario	Training Samples	Algorithm	RMSE
Early Prediction (EP)	50	ANN	$2.67\times 10^{-2}$
		LSTM	$2.31\times 10^{-2}$
		SVM	$1.81\times 10^{-2}$
		GPR	$1.34\times 10^{-2}$
		LSTM Proposed	$1.30\times 10^{-2}$
Mid-Life Prediction (MLP)	80	SVR	$8.10\times 10^{-3}$
		XGBoost	$1.95\times 10^{-2}$
		Linear NN	$1.20\times 10^{-2}$
		Lasso	$9.0\times 10^{-3}$
		Ridge	$1.30\times 10^{-2}$
		ElasticNet	$8.50\times 10^{-3}$
		GPR	$9.80\times 10^{-3}$
		LSTM Proposed	$7.69\times 10^{-3}$

## Data Availability

The raw data supporting the conclusions of this article will be made available by the authors on request.

## Abbreviations

The following abbreviations are used in this manuscript:

SOH	State of Health
RUL	Remaining Useful Life
LSTM	Long Short-Term Memory
ESS	Energy Storage Systems
ML	Machine Learning
DL	Deep Learning
RNN	Recurrent Neural Network
MSE	Mean Square Error
RMSE	Root Mean Square Error
EP	Early Prediction
MLP	Mid-Life Prediction
AP	Advanced Prediction
RDT	Raw Data Training
SEM	Single Exponential Model
